# Harmful alcohol consumption in women with mental illness a descriptive study in a psychiatric short stay unit

**DOI:** 10.1192/j.eurpsy.2025.990

**Published:** 2025-08-26

**Authors:** B. Samsó, A. López, U. Lopez, R. Lopez, M. Mendez, A. Darriba, M. Cabrera, C. Martinez, L. M. De alfonso, X. Fernandez, J. A. Baeyens, N. Rodriguez, M. Salinero, E. M. Garnica, A. Bilbao

**Affiliations:** 1 RED DE SALUD MENTAL DE BIZKAIA, Zamudio, Spain

## Abstract

**Introduction:**

Research suggests that the gender gap in alcohol (Alc.) consumption may be narrowing, with increasing consumption by the female population. For women, the equivalent of 20-25 grams/day is considered harmful consumption. Many of the harmful effects of alcohol consumption occur more rapidly and more severely in women, yet the majority do not seek treatment

**Objectives:**

To describe the prevalence of harmful use of alcohol in women admitted to hospital for other mental illness.

**Methods:**

Retrospective observational study of six months duration. Data was collected from all patients admitted to the hospital during the period of study, with no specific exclusion criteria. Descriptive statistics were performed.

**Results:**

During the study period, 312 patients were admitted to the hospital, 52.8% of whom were women, 21.8% of whom had a harmful use of Alc. The prevalence of harmful Alc. use in the different mental illnesses was: 22.2% in schizophrenia or other psychoses, 13.9% in bipolar affective disorder, 11.1% in depressive disorder, 13.9% in personality disorder, 19.4% in substance use disorder, 13.9% for other diagnoses and a prevalence of 5.56% for those of unknown diagnosis

**Conclusions:**

In recent years, it has been shown that the overall prevalence of harmful alcohol use and alcohol use disorder has increased in women. They tend to seek care in settings where their alcohol problems may not be recognised. They have lower social support for treatment participation and greater barriers to accessing treatment, resulting in even higher rates of co-occurring medical and mental health problems. Prevention strategies and intervention programmes should be adapted to the specific needs of women, taking into account both individual risk factors and socio-cultural contexts

**Disclosure of Interest:**

None Declared

